# Studying the effect of processing parameters on the microstructure, strength, hardness, and corrosion characteristics of friction stir dissimilar welded AA5083 and AA7075 aluminum alloys reinforced with Al-SiC matrix

**DOI:** 10.1016/j.heliyon.2024.e41362

**Published:** 2024-12-19

**Authors:** Noha M. Abdeltawab, Mohamed Elshazly, Ahmed Y. Shash, M. El-Sherbiny

**Affiliations:** a Department of Mechanical Design and Production, Faculty of Engineering, Cairo University, Giza, 12316, Egypt; b Faculty of Engineering and Materials Science, German University in Cairo, Cairo, Egypt

**Keywords:** Dissimilar friction stir welding, Aluminum silicon carbide matrix, Processing parameters, AA5083/AA7075 welding

## Abstract

The complementary properties of corrosion resistance and ballistic resistance of AA5083 and AA7075, respectively, explain the significance of welding these two alloys in the marine armor industry. This study investigates a novel Al-SiC matrix reinforcement with a different SiC weight ratio in dissimilar friction stir welding of the AA5083/AA7075 joint at different transverse and rotational speeds. The study deduced that the novel matrix can play an important role in improving strength and ductility simultaneously. Maximum strength takes place under conditions of 50 % SiC in Al-SiC matrix, 900 rpm rotational speed, and 40 mm/min transverse speed of value 209.8 MPa, which is equal to 90 % strength of the base metal AA5083, and the maximum strain takes place at 25 % SiC in Al-SiC matrix, 900 rpm rotational speed, and 20 mm/min transverse speed, with improved strain by 2 % than the softer alloy AA5083. Corrosion weight loss decreased by increasing the SiC weight ratio in the Al-SiC matrix, where it improved by 47.9 % compared to the base metal alloy of AA5083 at 75 % SiC in the Al-SiC matrix, 900 rpm rotational speed, and 20 mm/min transverse speed.

## Graphical abstract

**Figure:**
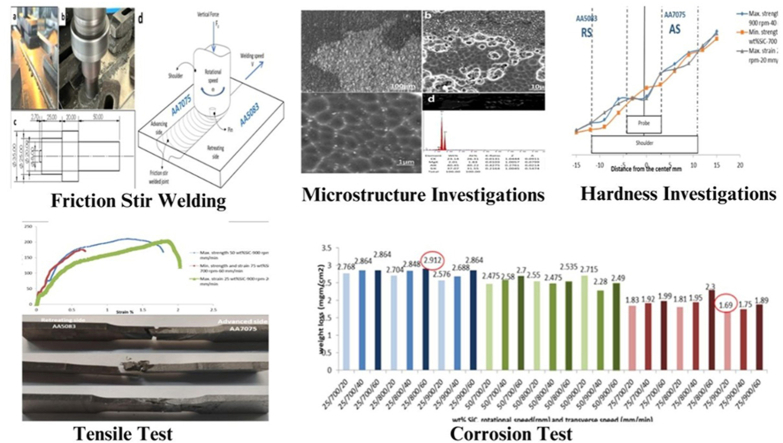

## 1 Introduction

The alloys of AA5083 and AA7075 alloys offers a number of promising and desirable properties, AA7075 has the advantage of ballistic resistance [1,2] while AA5083 is renowned for corrosion resistance that nominate the assembly structures to replace steel armor in many application like marine industry, aerospace, and automobiles [3,4]. Al-Zn-Mg-(Cu) (AA7xxx) alloys are a heat-treatable precipitation hardenable series [3,5]. The hardening isothermal process takes place in the sequence starting from a supersaturated solid solution followed by the forming of Guinier-Preston (GP) zones and η-phase along the grain boundaries [6]. AA7075 alloys have the advantage of a high strength-to-weight ratio, good ductility, energy absorption capacity, and high cryogenic properties [7]. However, applications of AA7075 face challenges of low corrosion resistance and the high content of Cu (1.75 wt%) susceptible the alloy to cracks and elevated residual stresses during fusion welding [8]. Additionally, the thermal cycle and severe plastic deformation welding processes like Friction Stir Welding FSW lead to dissolving the precipitates (ηʹ phases), softening, and reduction in mechanical properties [9,10]. AA5083 is an aluminum-magnesium alloy where the presence of secondary phases such as Al6Mn and Mg2Al3 plays critical roles in precipitation hardening as it obstacles dislocation motion and improves strength. Additionally, on account of the presence magnesium, AA5083 has high resistance to corrosion and stress corrosion cracking [11]. Fusion welding can adversely affect AA5083 through melting and solidification, causing brittle intermetallic compounds and grain growth [12].

FSW is a solid-state welding process where a rotating pin tool is imposed on the workpiece, causing high friction and induced heat that raises metal plasticity in the presence of stirring movement to merge the interfaces of the welding joint [13,14]. FSW has successfully overcome the drawbacks of fusion welding of aluminum alloys, such as cracks, distortion, and porosity inclusions [15]. Keeping the welding joint in solid states prevents melting and solidification problems like brittle dendritic structures, coarse grains, and eutectic phases that take place in fusion welding. Reinforcing FSW joints with nanoparticles allows controlling their microstructure by preserving the fine and equiaxed grains in the welding nugget zone, which enhance mechanical properties. The role that nanoparticles (SiC, Al2O_3_, SiO_2_, B_4_C, and TiC) play in improving the joint corrosive and wear resistance raises the concern of its applicability in FSW [16,17]. The silicon carbide (SiC) nanoparticles as compared to other reinforcements have a lower density and show high wettability and compatibility with aluminum alloys. The addition of SiC nanoparticles in FSW intercepts grain growth and dissolving (ηʹ phases) in AA7075 through the pinning effect as it pins the grain boundary movement during welding [13,18]. Previous results show that the tensile strength and elongation of AA7075-O were enhanced by up to 7.2 % and 137.7 %, respectively, with the addition of SiC nanoparticles. Compared to SiC free specimens, SiC reinforced specimens had higher levels of hardness and ductile fracture modes in the base material. On the other hand, in SiC free specimens, tensile fracture happened in the Thermo-Mechanically Affected Zone (TMAZ) since it did not pass through recrystallization [19]. Microstructure investigation of AA7075 friction stir welding reinforced by SiC shows that SiC reinforcements and aluminum matrix have a good bond where the enhancement in tensile strength, toughness, and fatigue life is due to grain refinement and effective load transfer across the matrix in the presence of uniform distribution of SiC nanoparticles [17].

The FSW process parameters of rotational speed, transverse speed, tool shape, and number of passes have a significant effect on the quality of the welding joint and the dispersion of SiC nanoparticles [13,18]. In an investigation of a similar AA7075/SiC-reinforced joint produced by FSW, it was found that as rotation speed increases, a good dispersion of SiC nanoparticles occurs, leading to an improvement in mechanical properties. Samples of higher rotational speed 1250 rpm have a uniform reinforcement distribution, and finer grains take place due to high stirring action [[20], [21], [22]]. In a study of dissimilar FSW joints of AA7075/AA6061 reinforced by SiC nanoparticles, results showed that at a low rotation speed of 700 rpm, inadequate dispersion and bonding of SiC nanoparticles were noted, reflecting poor mechanical properties compared with a higher rotation speed of 1100 rpm [23]. Tensile strength and hardness increase as transverse speed increases as a consequence of lower heat input, which leads to grains getting finer and the dissolution of hardening participants getting lower [24]. Microstructure investigation of AA5083 friction stir welding reinforced with SiC and TiC on the reinforcing nanoparticle distribution deduced that a uniform dispersion of nanoparticles is favored by a high number of processing passes along with low traversal and high rotating speeds [25]. The influence of process parameters in reinforced friction stir welding of AA5083 by TiO_2_ shows that the hardness and ultimate tensile strength after four passes with a rotational speed of 710 rpm and a forward speed of 14 mm/min were increased by 40 % and 25 % when compared to that of unreinforced AA5083 alloy [26].

Friction stir welding of AA5083 with AA7075 is significant for applications requiring a combination of strength, corrosion resistance, and ballistic resistance, like armor and marine industry. The exploration of novel reinforcements may assist in regulating heat input and material flow during welding, hence minimizing the formation of brittle phases. Reinforcements can also restrict the formation of intermetallic compounds that weaken the joints and reduce porosity and void development as Well-distributed reinforcements can assist fill voids and reduce the production of welding flaws. Reinforced the joint with the novel matrix of Al-SiC is supposed to improve wettability and compatibility between the joint and reinforced matrix at different rotational speed and transverse speed to enhance mechanical and corrosion properties.

## 2 Experimental procedure

### 2.1 Input material specification and experimental setting configuration

**Fig. 1:**
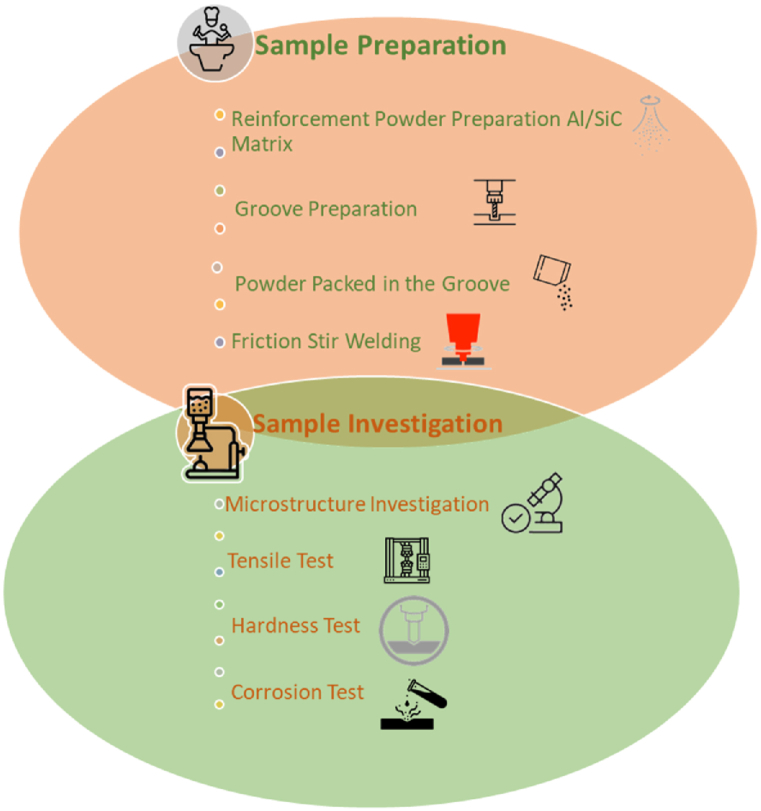
Flow of experimental work.

In this paper, the flow of experimental work is illustrated in Fig. 1. A strip of AA5083 and AA7075 of equal dimension (200 × 50 × 3 mm3) was used as the substrate for FSW. Table 1 shows the chemical composition of the samples for each alloy measured by the spectrometer. The mechanical properties of the two alloys of base material were tested and mentioned in Table 2. To prepare the reinforcement matrix powder, mechanical powder ball milling takes place of ALPOCO© aluminum powder and SiC with particle size 25, 2 μm, respectively, mixed with weight percent wt.% of silicon carbide 25 wt%, 50 wt%, and 75 wt%. The powder mixture undergoes a total of 6 h of mechanical powder ball milling with a ball-to-powder ratio of 5:1 and a milling speed of 400 rpm to ensure good dispersion and complete encapsulation of SiC particles with aluminum.

**Table 1:** Chemical composition of AA5083 and AA7075.

AA5083 Chemical Composition								
Element	Al	Mg	Mn	Fe	Si	Zn	Ti	Cu
Content	Bal.	4.2	0.59	0.38	0.39	0.15	0.1	0.08

AA7075 Chemical Composition								

Element	Al	Mg	Mn	Cr	Si	Zn	Ti	Cu
Content	Bal.	2.8	0.25	0.21	0.2	5.92	0.1	1.8

**Table 2:** Mechanical properties of AA5083 and AA7075.

AA5083 Mechanical Properties				
Property	Maximum Strength(Mpa)	Maximum Strain %	Hardness (HV)	Corrosion Weight loss (mgm/cm^2^)
Value	230	1.97 %	85	2.5

AA7075 Mechanical Properties				

Property	Maximum Strength(Mpa)	Maximum Strain %	Hardness (HV)	Corrosion Weight loss (mgm/cm^2^)
Value	505	1.35	220	3.5

**Fig. 2:**
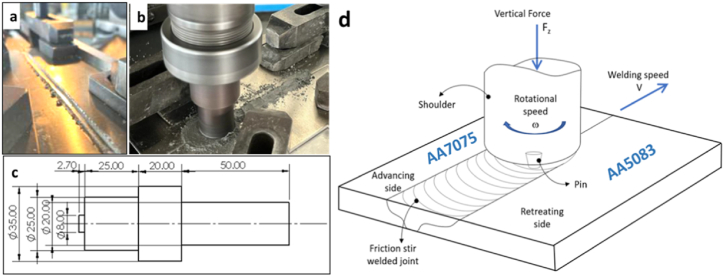
Friction stir welding process (a) groove preparation, (b) packing powder in the groove, (c) pin tool dimension and (d) experimental setting of plates and welding rotational and transverse direction.

A shown in Fig. 2-a, To prepare the welding joint, a groove of 3 mm in width and 2.5 mm in depth was machined across the center of the two substrates along the length of the samples. The reinforcement powder was packed in the groove using a pin-less tool of 25 mm in diameter, as shown in Fig. 2-b. A conventional vertical milling machine was used in the friction stir welding process, where the pin tool, which is made off K110 tool steel, has a detailed dimension shown in Fig. 2-c. The experimental setting configuration, rotational and transverse speed direction, is shown in Fig. 2-d. To ensure effective bonding, the harder alloy of AA7075 is placed on the advanced side, as it needs more heat due to the collective influence of the rotational and translational speed of the tool. However, the more ductile alloy AA5083 is placed on the retreating side to attain a balanced temperature distribution through the welding joint [23]. The values for the process parameters are determined based on previous research, the experimental capabilities that are available, and trial experiments carried out on AA5083 and AA7075. In these experiments, the L27 orthogonal array, whose rotation speed varies from 700, 800, and 900 rpm and transverse speed is 20, 40, and 60 mm/min as shown in Table 3, three repetitions was used as the basis for design of experimental settings.

**Table 3:** L27-OA of experimental investigation.

Run Number	SiC % wt in Al-SiC Matrix	Rotational Speed(rpm)	Transverse Speed(mm/min)
1	25	700	20
2	25	700	40
3	25	700	60
4	25	800	20
5	25	800	40
6	25	800	60
7	25	900	20
8	25	900	40
9	25	900	60
10	50	700	20
11	50	700	40
12	50	700	60
13	50	800	20
14	50	800	40
15	50	800	60
16	50	900	20
17	50	900	40
18	50	900	60
19	75	700	20
20	75	700	40
21	75	700	60
22	75	800	20
23	75	800	40
24	75	800	60
25	75	900	20
26	75	900	40
27	75	900	60

### 2.2 Microstructure investigation

To examine the microstructure of the sample, an optical microscope and a Zeiss LEO SUPRA 55-Field Emission Scanning Electron Microscope (SEM, Selangor, Malaysia) were utilized, and the joint surface was analyzed by means of energy dispersive X-ray spectroscopy (EDX) within the SEM.

### 2.3 Vickers hardness test

Vickers hardness measurement was applied to welding nugget cross-section polished samples. A microhardness HV-50A (Vickers, London, UK) tester is used to assess hardness under 10 N load applied and 11 S dwell duration. Eleven readings along the welding joint, ranging from the center to the base material, yield the results that are presented.

### 2.4 Tensile test

**Fig. 3:**
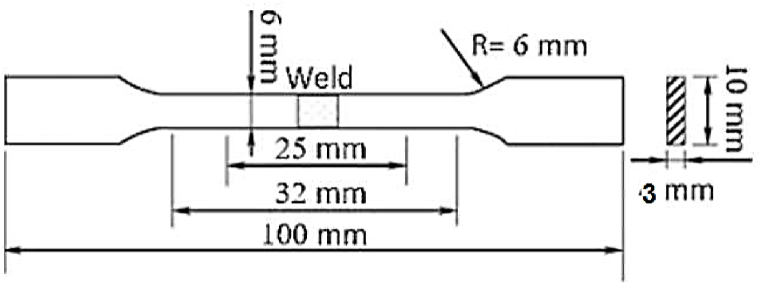
Tensile sample dimension ASTM E8 standards.

Using a wire cutting process, tensile specimens were trimmed out perpendicular to the welding zone in accordance with precise dimensions that complied with ASTM E8 [27], as illustrated in Fig. 3. Universal tensile testing equipment is used for uni-axial tensile testing at ambient temperature. The average of three samples from three distinct welded plates was used as the basis for all results.

### 2.5 Corrosion test

In order to evaluate the corrosion rate using immersion test method by weight loss, the samples were cut to the dimensions of 10 mm × 10 mm × 3 mm. The weights of the samples were measured before immersion, and the samples were immersed in a 3.5 % NaCl solution. To remove the corrosion outcomes, a solution of 50 g chromium trioxide (CrO_3_), 2.5 g silver nitrate (AgNO_3_), and 5 g barium nitrate (Ba(NO_3_)_2_) in 250 ml distilled water was utilized. Following that, the weight of the samples was measured, and the net weight loss was evaluated according to ASTM G31-72 criteria, as weight loss per lateral surface area [28].

## 3 Results and discussion

L27 orthogonal array experimental settings were carried out according to Table 3. Overall, it could be observed that joint strength and quality are highly affected by the presence of reinforcement matrix, rotational speed, and transverse speed, where the welding observations are mentioned in Table 4. Samples at the highest rotational speed of 900 rpm and low transverse speed of 20 mm/min suffer from flash defects along the edges of the weld seam that could extend to thickness reduction at low SiC wt.% in the Al-SiC matrix; however, as SiC wt.% increases, it changes to minor burr formation, which is an indication of good weld consolidation, material flow, and mixing [29]. At the high transverse speed of 60 mm/min and mostly when it is combined with low rotational porosity, cracks and voids appear in the cross-section of the welding joint that extend to high brittleness as it serves as initiation points for fracture propagation. This contemplates the importance of balancing and tailoring between parameters that affect the mixing and material flow experimentally or by numerical modeling and optimization [30].

**Table 4:** Tensile test results.

No	SiC % wt in Al-SiC Matrix	Rotational Speed (rpm)	Transverse Speed (mm/min)	Ultimate Tensile Strength Mpa	Max. Strain	Observations	Fracture Zone
1	25	700	20	191.4	1.85	flash defect and reduction in thickness	Weld metal + ductile fracture
2	25	700	40	186.2	1.75	Flash defect	Weld metal + ductile fracture
3	25	700	60	179.7	1.7	voids, cracks	Weld metal + ductile fracture
4	25	800	20	196.6	1.86	flash defect and reduction in thickness	Weld metal + ductile fracture
5	25	800	40	189.4	1.85	Prefect welding	Weld metal + ductile fracture
6	25	800	60	187.1	1.79	Voids and cracks	Weld metal + ductile fracture
7	25	900	20	201.4	2.01	Flash defect reduction in thickness	Weld metal + ductile fracture
8	25	900	40	198.8	1.94	Flash defect	Weld metal + ductile fracture
9	25	900	60	191.5	1.87	Minor voids and porosity can be detected in welding cross section	Weld metal + ductile fracture
10	50	700	20	197.9	1.6	Prefect welding	Weld metal + ductile fracture
11	50	700	40	195.3	1.52	Minor voids and porosity can be detected in welding cross section	Weld metal + ductile fracture
12	50	700	60	191.3	1.48	voids, cracks	Weld metal + ductile fracture
13	50	800	20	203.6	1.62	Prefect welding	Weld metal + ductile fracture
14	50	800	40	198.4	1.56	Prefect welding	Weld metal + ductile fracture
15	50	800	60	194.9	1.48	Minor voids and porosity can be detected in welding cross section	Weld metal + ductile fracture
16	50	900	20	209.8	1.67	Prefect welding + formation of minor burrs	Weld metal + ductile fracture
17	50	900	40	200.8	1.62	Prefect welding	Weld metal + ductile fracture
18	50	900	60	194.8	1.52	Minor voids and porosity can be detected in welding cross section	Weld metal + ductile fracture
19	75	700	20	184.7	0.93	voids, cracks	AA5083 interface + brittle conchoidal
20	75	700	40	183.5	0.85	voids, cracks	Weld metal + brittle conchoidal
21	75	700	60	175.3	0.68	voids, cracks	AA5083 interface + brittle conchoidal
22	75	800	20	191.4	0.9	Prefect welding	Weld metal + brittle conchoidal
23	75	800	40	183.5	0.89	voids, cracks	AA5083 interface + brittle conchoidal
24	75	800	60	179.6	0.72	voids, cracks	Weld metal + brittle conchoidal
25	75	900	20	196.7	1.2	Prefect welding + formation of minor burrs	Weld metal + ductile fracture
26	75	900	40	188.9	0.9	voids, cracks	Weld metal + brittle conchoidal
27	75	900	60	183.3	0.78	Minor voids and porosity can be detected in welding cross section	AA5083 interface + brittle conchoidal

### 3.1 Microstructure investigation

**Fig. 4:**
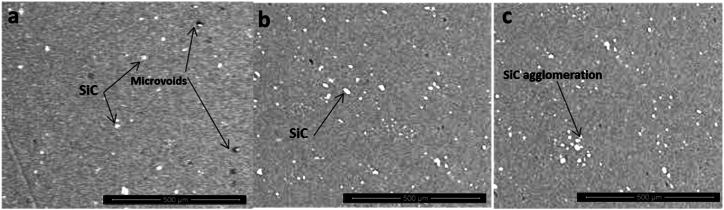
Microstructure of Welding Joint a) Sample 7 Under Condition 25 % SiC wt.% in Al-SiC, 900 rpm, 20 mm/min, b) Sample 16 Under Condition 50 % SiC wt.% in Al-SiC, 900 rpm, 20 mm/min and c) Sample 25 Under Condition 75 % SiC wt.% in Al-SiC, 900 rpm, 20 mm/min.

**Fig. 5:**
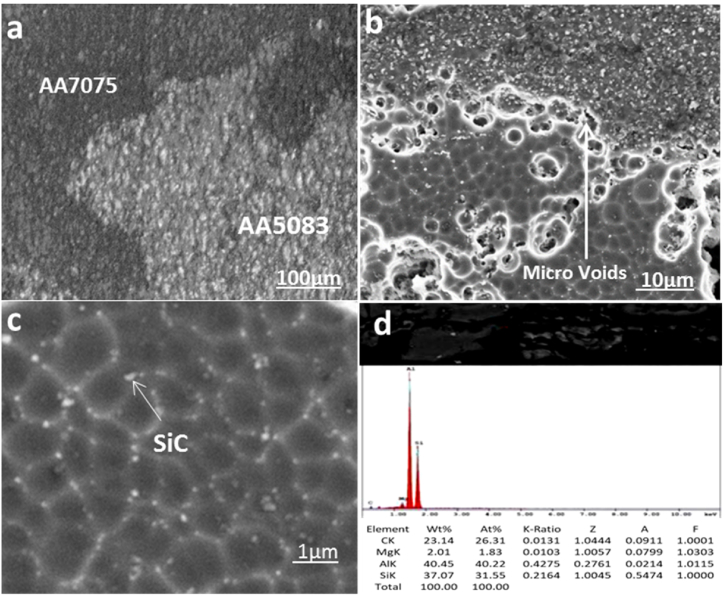
SEM Microstructure of Sample 16 Under Condition 50 % SiC wt.% in Al-SiC, 900 rpm and 20 mm/min, a)Interface Between The Two Alloys, b) Further Magnification, C) AA5083 alloy, d)EDX Investigation.

Cross-section micrograph investigation by SEM of reinforcement joint with different SiC wt.% in Al-SiC matrix at contestant rotational speed and transverse speed of 900 rpm and 20 mm/min, respectively. Fig. 4-a shows the microstructure of sample 7 with 25 % SiC in the Al-SiC matrix. While the SiC shows uniform dispersion, the sample has some microvoids that increase the potential for concentrated stresses. On the other hand, sample 16, which has the highest strength, as shown in Fig. 4-b, has good dispersion of SiC in the joint, which improves the load-transfer efficiency and enhances the mechanical properties of the joint, consistent with previous studies [18]. Increasing SiC wt.% in Al-SiC to 75 % in Fig. 4-c shows agglomeration of SiC particles that decrease tensile strength as they work as localized stress concentration points, and inhomogeneity reduces load-bearing capacity [20]. A further magnification is shown in Fig. 5-a for sample 7 in the nugget zone, where it shows the interface between the AA7075 and the nugget zone with overlap, and the onion shape ensures good cohesion in the welding joint [18]. The advancing side of the AA7075 alloy has a more refined grain structure because of the high plastic deformation and heat generation, which supports dynamic recrystallization. Fig. 5-b shows mixing bands of the grain gradient between the nugget zone as well as the presence of microvoids and porosity in the interface that stabs the structural integrity. In Fig. 5-c, SiC particles can be seen along the grain boundaries in the nugget zone that work in grain refinement and improve mechanical properties. It confirms what has been proposed in previous studies [19]. The EDX investigation for the nugget zone in Fig. 5-d confirms the presence of SiC on the grain boundary that can improve joint phase distribution, decreasing the formation of agglomeration of intermetallic compounds that would decrease ductility while the existence of Mg is indication of formulation of Al-Zn-Mg-Cu intermetallic compound.

**Fig. 6:**
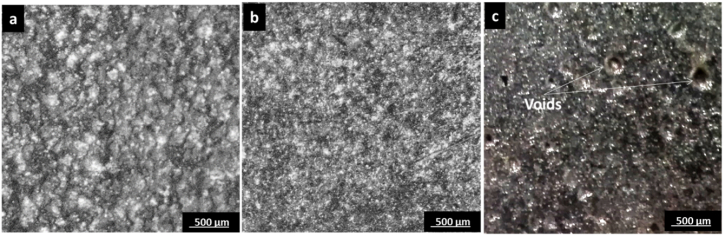
Microstructure Investigation Under Different Transverse Speed a) Sample 13 Under Condition 50 % SiC wt.% in Al-SiC, 800 rpm, 20 mm/min, b) Sample 14 Under Condition 50 % SiC wt.% in Al-SiC, 800 rpm, 40 mm/min and c) Sample 15 Under Condition 50 % SiC wt.% in Al-SiC, 800 rpm, 60 mm/min.

**Fig. 7:**
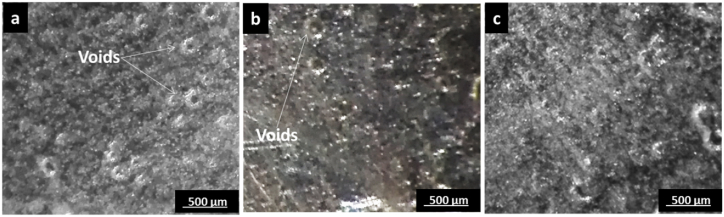
Microstructure Investigation Under Different Rotational Speed a) Sample 11 Under Condition 50 % SiC wt.% in Al-SiC, 700 rpm, 40 mm/min, b) Sample 14 Under Condition 50 % SiC wt.% in Al-SiC, 800 rpm, 40 mm/min and c) Sample 17 Under Condition 50 % SiC wt.% in Al-SiC, 900 rpm, 40 mm/min.

In cross-section micrographs and optical microscope investigations of samples, it could be noted that the absence of any falling or remaining reinforcement filler is an indication of its complete dissolution and mixing in the base material. The effect of different transverse speeds is illustrated in Fig. 6 at constant studied parameters of 50 % SiC wt.% in the Al-SiC matrix and rotational speed of 800 rpm. Fig. 6-a shows sample 13 at a transverse speed of 20 mm/min, where it has relative coarser grains due to high heat input generated and a low cooling rate at a low transverse speed, while in Fig. 6-b the grain is finer at a transverse speed of 40 mm/min. At the highest transverse speed in Fig. 6-c, the presence of porosity is noted, and a smaller grain size. This may take place due to trapped gases during the welding process. This may come as a result of the way the reinforcement filler was packed in the groove or insufficient heat to mix the boundaries between joints at high transverse speed, which is consistent with the importance of previously studied thought-out of the transverse speed [23]. Furthermore, the AA7075 alloy requires higher heat input for proper plasticization, while the AA5083 and the reinforcement matrix are overheated. This irregular heat distribution can cause poor mixing and insufficient bonding, forming voids or trapped gases. Cross-section micrographs and optical microscope investigation of samples at different rotational speeds is illustrated in Fig. 7 and at constant 50 % SiC weight in the Al-SiC matrix and transverse speed of 40 mm/min. At the low rotational speed of 700 rpm in Fig. 7-a, porosity and microvoids can be noted due to poor material flow, and the stirring effect at low rotational speed leads to incomplete bonding. Furthermore, inadequate stirring of the AA7075, due to its higher strength and lower plasticity, results in voids or porosity in the weld nugget. However, at higher rotational speeds in Fig. 7-b and 7-C, the porosity and void defect decrease, which is consistent with previous analyses [31].

### 3.2 Tensile Test Results

**Fig. 8:**
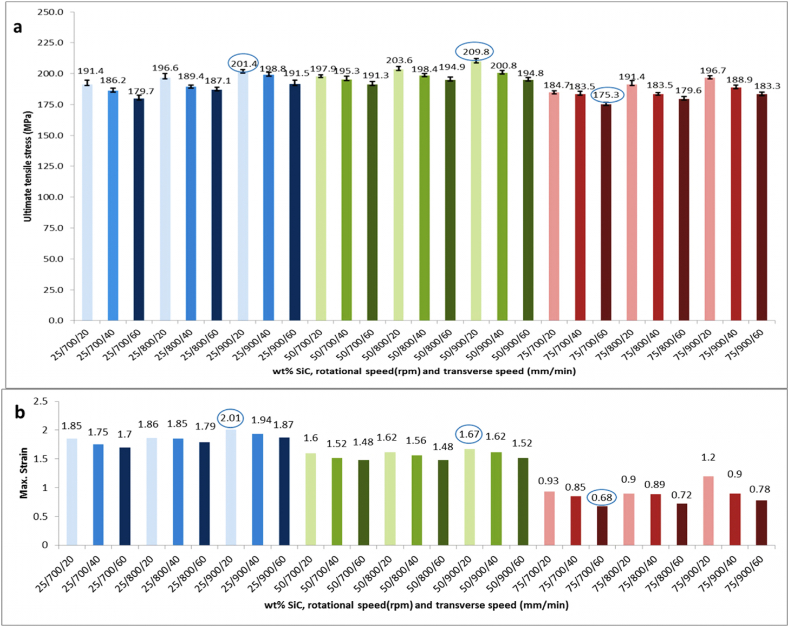
Tensile Test Results a) Ultimate Tensile Strength, b) Maximum Strain.

Over all, the ultimate tensile strength value of the welding joint is from 70 % to 90 % of the AA5083 base alloy strength, which is the softer material that dominates the performance of the joint, which is an indication of a successful welding process. Similarly, the maximum strain value is close to the softer alloy AA5083, and the fracture takes place in the nugget zone at the interface between the weld and the AA7075, as mentioned in Table 4. It is consistent with previous tests [32]. It could be noted that the novel reinforcement of the Al-SiC matrix improves the ductility and maximum strain of the joint and overcomes the problem of embrittlement that takes place in the AA5083/AA7075 joint [32] and SiC-AA5083/AA7075 reinforcement joint in previous research [33]. Fig. 8-a shows the ultimate strength of the welding joint, where it can be deduced in general terms that as transverse speed decreases and fixing the other parameters, the ultimate strength increases. As well the ultimate tensile is increase in fixing other parameters and increasing the rotational speed. There are one or two results that do not correspond to the aforementioned behavior. This general behavior is consistent with previous results and microstructure analyses, which, by means of reducing the transverse speed, the time for joint interaction in the stir zone increases, which gives sufficient plasticization and mixing of materials [33]. Otherwise, reducing rotational speed reduces the stirring effect, leaving gaps that can generate porosity, voids, and cracks, and the joint failed to properly consolidate the two alloys, as illustrated in the previous microstructure investigation. Fig. 8-b demonstrates the relationship between wt% SiC, rotational and transverse speed, and their effects on the material's maximum strain. Average wt% SiC values (50 %) result in considerable strain values, ranging from 1.48 to 1.94 depending on rotational and transverse speeds. High-speed conditions with a higher SiC content (75 %) appear to result in lower strain values, implying decreased material ductility or increased brittleness under high reinforcement and processing speeds.

**Fig. 9:**
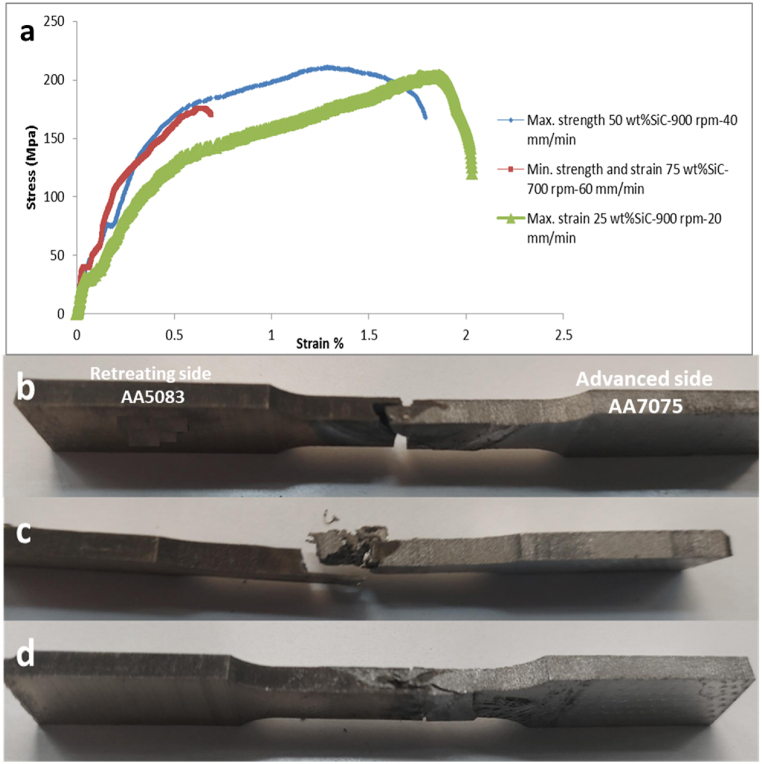
a) Stress Strain Curve of Maximum Strength, Minimum Strength and Strain, Maximum Strain Sample (b) Tensile Fracture of Maximum Strength Sample 16 (c) Tensile Fracture of Minimum Strength and Strain Sample 21 (d) Tensile Fracture of Maximum Strain Sample 7.

Fracture zone and mode in the tensile test mentioned in Table 4 of friction stir welding give an important indication of the welding quality that is governed by reinforcement dispersion and a tailored balance of rotational and transverse speeds. Fracture takes place in weld metal refers to the zone where the joint experiences stress and strain, ultimately. Ductile fracture in weld metal and a strength value close to AA5083 alloy strength denote that the softer alloy AA5083 is the limiting factor for the weld's overall strength. While brittle fracture in weld metal is a high indication of cracks and voids that work as localized stress points. The stress-strain curve of the critical samples of maximum strength, minimum stress, strain, and maximum strength is shown in Fig. 9-a. In Fig. 9-b, the maximum strength sample 16 is shown under conditions of 50 % SiC in the Al-SiC matrix, 900 rpm rotational speed, and 40 mm/min transverse speed. Sample 16 has fracture ductile mode with a dimpled surface in the weld metal zone, which is an indication of good reinforcement dispersion and a good balanced rotation and transverse speed. In Fig. 9-c, fracture of sample 21 of minimum strength and strain is shown at conditions of 75 % SiC in the Al-SiC matrix, 700 rpm rotational speed, and 60 mm/min transverse speed. It has a crumbly fracture in the AA5083 interface, which is an indication of the presence of voids and cracks that work as initial cracks and help in crack propagation. This takes place at the highest SiC wt.%. and may face agglomeration in combination with low rotational speed and high transverse speed. On the other hand, sample 7 of the maximum strain in Fig. 9-d shows ductile fracture mode in the weld metal with a very good mixing situation at 25 % SiC in the Al-SiC matrix, 900 rpm rotational speed, and 20 mm/min transverse speed. From the results, it can be deduced that the presence of the novel reinforcement matrix can improve strength and ductility through orowan strengthening and grain refinement at a good balance of reinforcement matrix dispersion and a balance between rotational speed and transverse speed that goes with previous research deductions [33].

**Fig. 10:**
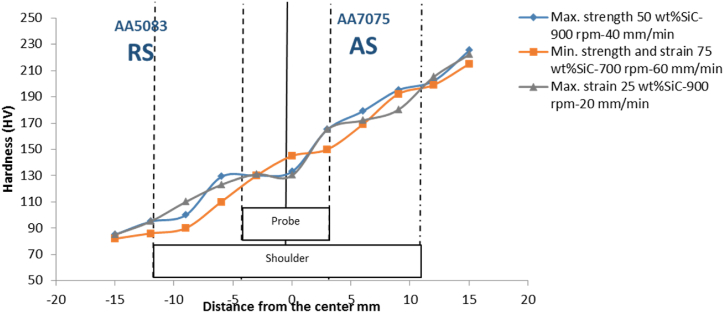
Hardness results of critical samples.

The hardness of dissimilar materials varies dramatically between these zones because each material responds differently to the FSW process. Hardness profiles aid in figuring out how alloys interact during the process, particularly metallurgical compatibility, developing intermetallic compounds, distributing thermal and mechanical stress, and dispersing reinforcement [34]. The hardness distribution across the welding joint in samples of maximum strength, maximum strain, and minimum strength and strain is shown in Fig. 10, where the hardness value is increasing gradually from the base metal of AA5083 to the welding metal of AA7075. That reflects the effect of mechanical processing, grain refinement, good dispersion of reinforcement matrix, and formation of intermetallic compounds to improve the weld joint hardness over the soft base metal in AA5083. As well, the heat-affected zone at the center of the welding has the highest hardness value in the hardness distribution only on the sample of minimum strength and strain that takes place at the highest transverse speed and lower rotational speed, which is an indication of insufficient heat input and a high cool rate under that condition that leads to hardening and the presence of residual stresses [33].

**Fig. 11:**
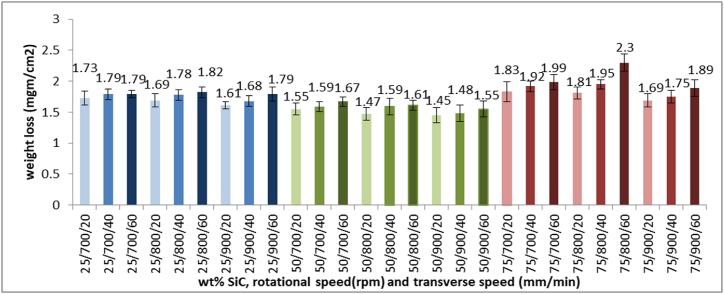
Corrosion Weight Loss of Samples with Different wt. % SiC, Rotational Speed and Transverse Speed.

Corrosion behavior is affected by microstructural changes that take place in reinforcement, dissimilar friction stir welding of grain refinement, residual stresses, and intermetallic compounds. Comparing to the base material, the overall total corrosion weight loss per centimeter squared at 90 days is lower than the results shown in Fig. 11, which is an indication of the good impact of reinforcement Al-SiC matrix and grain refinement due to mechanical processing to improve the corrosion properties of the joint, which is a repetitive deduction in previous research [35]. Over all, it can be deduced that increasing SiC wt.% in the Al-SiC matrix with the other parameters installed improves corrosion behavior as its grain refinement effect decreases the amount of grain boundaries that work as corrosion initiation locations. As well, fixing other parameters and increasing the rotational speed improve reinforcement dispersion and increase the formation of intermetallic compounds that barrier joint exposure to the corrosive solution. The lowest corrosion weight loss takes place in sample 25 at 75 % SiC in the Al-SiC matrix, 900 rpm rotational speed, and 20 mm/min transverse speed. High transverse speed resulted in coarse grains that form locations of differing corrosion potential that increase corrosion. The highest corrosion weight loss takes place in sample 6 at 25 % SiC in the Al-SiC matrix, with an 800 rpm rotational speed and 60 mm/min transverse speed that alert the integration effect of rotational and transverse speed on corrosion resistance to balance the microstructure of the produced joint.

## 4 Conclusion

The novel reinforcement matrix of Al-SiC with different SiC wt.% was applied to AA5083/AA7075 dissimilar friction stir welding at different rotational and transverse speeds. A microstructure investigation, tensile test, hardness test, and corrosion test were conducted to study the effect of these different parameters and deduce that.

• The novel reinforcement of the Al-SiC matrix at balanced rotational and transverse speeds can improve both ductility and strength of joints through grain refinement and reduction of stress concentration points. As well as decreasing corrosion weight loss due to grain refinement, reduces the quantity of grain boundaries that serve as sites for the start of corrosion.
• Compared to base material, the maximum welding joint ultimate strength is 90 % of AA5083 ultimate strength (the softer side), which is accepted and expected range in friction stir welding, while in favor of the presence of an reinforcement matrix, maximum strain is improved by 2 %. Corrosion weight loss improved by 47.9 % and 107.1 % compared to the base alloys AA5083 and AA7075, respectively, due to the reinforcement matrix, the formation of intermetallic compounds, and grain refinement.
• The maximum strength takes place at 209.8 MPa at sample 16 under conditions of 50 % SiC in the Al-SiC matrix, 900 rpm rotational speed, and 40 mm/min transverse speed. Sample 21 of minimum strength and strain of value 175.3 MPa and 0.68, respectively, conditions of 75 % SiC in an Al-SiC matrix, 700 rpm rotational speed, and 60 mm/min transverse speed. Sample 7 of maximum strain of value 2.01 at 25 % SiC in Al-SiC matrix, 900 rpm rotational speed, and 20 mm/min transverse speed.
• Tailoring the proper parameters of SiC wt% in the Al-SiC matrix, transverse speed, and rotational speed can improve tensile strength, maximum strain, and corrosion weight loss by 20 %, 195.5 %, and 72.3 %, respectively, which reflect the significance of the studied parameters in mixing the dissimilar alloys and grain refinement.
• The minimum weight loss of 1.69 mgm/cm2 takes place in sample 25 at 75 % SiC in Al-SiC matrix, 900 rpm rotational speed, and 20 mm/min transverse speed, and the maximum weight loss takes place at sample 6 at 25 % SiC in Al-SiC matrix, 800 rpm rotational speed, and 60 mm/min transverse speed.

Microstructural investigation shows good dispersion of SiC particles in a 50 % wt.% Al-SiC matrix that has higher effectiveness in strength and hardness results. At low rotational speeds, the mixing effect is reduced, and samples show porosity, voids, and even cracks if combined with low transverse speed, which leads to lower strength. At low transverse speed, insufficient heat input takes place, and microstructural investigation finds coarse grain and embrittlement that decrease the strength and strain.

## CRediT authorship contribution statement

**Noha M. Abdeltawab:** Writing – original draft, Visualization, Validation, Resources, Investigation, Formal analysis. **Mohamed Elshazly:** Visualization, Validation, Supervision. **Ahmed Y. Shash:** Writing – review & editing, Visualization, Validation, Supervision, Resources, Methodology, Investigation, Formal analysis, Conceptualization. **M. El-Sherbiny:** Writing – review & editing, Visualization, Validation, Supervision, Investigation.

## Data availability

Data will be made available on request.

## Declaration of competing interest

The authors declare that they have no known competing financial interests or personal relationships that could have appeared to influence the work reported in this paper.
